# Fluorogenic Real-Time Reporters of DNA Repair by MGMT, a Clinical Predictor of Antitumor Drug Response

**DOI:** 10.1371/journal.pone.0152684

**Published:** 2016-04-01

**Authors:** Andrew A. Beharry, Zachary D. Nagel, Leona D. Samson, Eric T. Kool

**Affiliations:** 1 Department of Chemistry, Stanford University, Stanford, CA, 94305, United States of America; 2 Departments of Biological Engineering and Biology, Massachusetts Institute of Technology, Cambridge, MA, 02139, United States of America; University of South Alabama Mitchell Cancer Institute, UNITED STATES

## Abstract

Common alkylating antitumor drugs, such as temozolomide, trigger their cytotoxicity by methylating the O6-position of guanosine in DNA. However, the therapeutic effect of these drugs is dampened by elevated levels of the DNA repair enzyme, O^6^-methylguanine DNA methyltransferase (MGMT), which directly reverses this alkylation. As a result, assessing MGMT levels in patient samples provides an important predictor of therapeutic response; however, current methods available to measure this protein are indirect, complex and slow. Here we describe the design and synthesis of fluorescent chemosensors that report directly on MGMT activity in a single step within minutes. The chemosensors incorporate a fluorophore and quencher pair, which become separated by the MGMT dealkylation reaction, yielding light-up responses of up to 55-fold, directly reflecting repair activity. Experiments show that the best-performing probe retains near-native activity at mid-nanomolar concentrations. A nuclease-protected probe, NR-1, was prepared and tested in tumor cell lysates, demonstrating an ability to evaluate relative levels of MGMT repair activity in twenty minutes. In addition, a probe was employed to evaluate inhibitors of MGMT, suggesting utility for discovering new inhibitors in a high-throughput manner. Probe designs such as that of NR-1 may prove valuable to clinicians in selection of patients for alkylating drug therapies and in assessing resistance that arises during treatment.

## Introduction

Alkylating agents are used as chemotherapeutic drugs to treat multiple cancers, including gliomas, melanoma, and Hodgkin’s disease [1]. These drugs induce cytotoxicity by forming covalent adducts with DNA. For example, temozolomide, a drug commonly used to treat glioblastoma, is a prodrug that spontaneously breaks down to form a methyldiazonium cation [2]. This highly reactive cation methylates multiple sites on DNA, including the N3-position of adenine and the N7 and O6 positions of guanine [2]. Among these alkylated products, cytotoxicity is mainly mediated by methylation at guanine *O*^6^ in DNA (*O*^6^-MedG). Polymerases misincorporate T opposite *O*^6^-MedG during DNA replication to generate a *O*^6^-MeG-T mismatch, which frequently leads to mutations and cellular dysfunction, and can ultimately lead to cancer cell death because of repeated unproductive processing by the DNA mismatch repair pathway [3,4].

A limiting factor in the effectiveness of drugs such as temozolomide is the development of tumor resistance. It is well established that resistance can result from upregulation of the repair protein *O*^6^-methylguanine-DNA-methyltransferase (MGMT, also denoted AGT) [5]. MGMT is a DNA repair protein that employs a cysteine residue to attack the *O*^6^-methyl group directly, leaving behind repaired guanine. As a result of this action, the protein is permanently alkylated (inactivated) and is marked for proteasomal degradation. MGMT is often overexpressed in tumors, particularly after an initial round of treatment, resulting in the reversal of the *O*^6^-alkylation and dampening the therapeutic effect of alkylating drugs [1, 6]. Alkylating drugs are therefore more effective in cancers that have low levels of MGMT, and for this reason, MGMT itself has become attractive as a drug target, since depleting its activity enhances the potency of alkylating drugs [1,7]. Thus, the level of activity of the MGMT repair system is important in diagnosis, prognosis and treatment of cancer, particularly for patients with brain tumors and melanomas [6]. In addition, temozolomide has shown significant therapeutic promise in recent clinical studies of acute myeloid leukemia (AML), a finding that also requires knowledge of MGMT activity [8]. Measuring MGMT repair activity in these diseases is valuable in predicting patient responses to alkylating drugs prior to treatment, guiding personalized therapies; in addition, monitoring activity over the course of therapy can be beneficial to evaluate any acquired resistance to the drug [1, 5].

Common methods to measure MGMT activity are indirect, complex and time-consuming. The most widely adopted approach is to use methylation-specific PCR to measure the degree of promoter methylation of CpG sequences for the *MGMT* gene [5,9]. However, due to its indirect nature, DNA methylation status correlates only moderately well with MGMT activity [10]. This weak correlation leads to difficulties in predicting resistance to anticancer alkylating drugs [10]. Another approach has used antibodies to quantify MGMT in an ELISA-based assay [11]. However, this method requires extensive washing steps and measures the amount of protein, not activity. Another common method uses a radiolabeled DNA substrate, whereby the degree of repair can be followed by transfer of the radioactive methyl group to the MGMT protein using procedures to separate protein and DNA [9,12]. Alternatively, ^32^P-labeled or fluorophore-labeled dsDNA probes containing the *O*^6^-MedG substrate have been employed; upon repair, a restriction enzyme cleavage site is generated and the degree of repair is followed by detection of DNA fragments by gel electrophoresis [13,14]. Although these methods do measure protein activity, the numerous steps required post-repair and the requirement for gel electrophoresis limit their use for rapid and/or high throughput assessments of MGMT activity. In this regard, a few fluorescence-based assays have been developed, aiming for simple and fast detection of MGMT. For example, Tintoré et al. [15] used an aptamer containing an *O*^6^-MedG lesion, as well as a fluorophore and a quencher at opposite ends of the sequence. A quadruplex structure is formed only upon MGMT repair, which results in a decrease in fluorescence as the fluorophore and quencher are brought in closer proximity. Although the assay does not require gel electrophoresis or isolation of reaction products, the reported signal changes were small (<2-fold), and measurements in whole cell lysates were not demonstrated [15]. Robinson et al. [16] developed an assay using a fluorophore labeled-O^6^-benzylguanine pseudosubstrate, whereby incubation with MGMT results in transfer of the fluorophore to the protein. Detection in whole cell lysates was demonstrated; however, separation of unreacted probe by thin layer chromatography was required for quantifying activity [16].

As a result of these limitations, and because of the clinical importance of measuring MGMT activity, a simple fluorescence-based assay that rapidly reports directly on activity with high-signal to noise within cell extracts could serve as a valuable tool for researchers and clinicians associating MGMT with cancer prognosis and treatment.

## Results

### Design and Synthesis of Fluorogenic MGMT Probes

**Fig 1:**
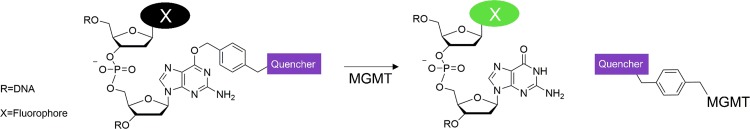
Design principle for a DNA-based chemosensor that reports on MGMT activity. A short modified DNA oligomer (see Table 1) contains an *O*^6^-benzylguanine nucleoside modified with a quencher dye. Fluorescence from a neighboring fluorophore (X) is initially quenched as a result of the proximity. When MGMT repairs the alkylated base, the benzyl-quencher group is transferred to the enzyme’s active site and fluorescence emission increases.

To design probes that can report on MGMT activity, we first focused on a developing a strategy for coupling fluorescence to the bond-breaking step that occurs during repair of O^6^-alkylguanine. Previous work on MGMT has established that the enzyme can repair an alternative substrate, O^6^-benzylguanine (BG), within a DNA oligonucleotide [17]. Notably, repair activity was found to be more efficient with BG than with its native *O*^6^-methylguanine substrate; the repair mechanism is the same except that the larger benzyl group is transferred to the MGMT active site. Subsequent studies have shown that modifications on the benzyl moiety, in particularly at the *para* position, do not alter enzymatic activity significantly [18]. With this in mind, we envisioned that a short DNA oligomer containing the BG substrate but synthetically modified on the benzyl group with a quencher (Fig 1), might still act as a good substrate for MGMT and simultaneously as a quencher for a neighboring fluorophore. In the presence of MGMT, the substrate would be expected to transfer its benzyl-quencher group to the MGMT active site, separating it from the fluorophore, and resulting in a fluorescence light-up signal from the repaired probe. Since the fluorescence signal is produced stoichiometrically with repaired guanine, such a probe would directly and quantitatively respond to MGMT activity in real time. It should be noted that while SNAP-tag probes (employing fluorophore-modified BG) have been developed that also transfer modified benzyl groups to a protein, they bind poorly to wild-type MGMT [19]. Based on the above reports, we reasoned that repair of BG within a DNA oligomer would result in strong binding and fast repair by native MGMT.

**Fig 2:**
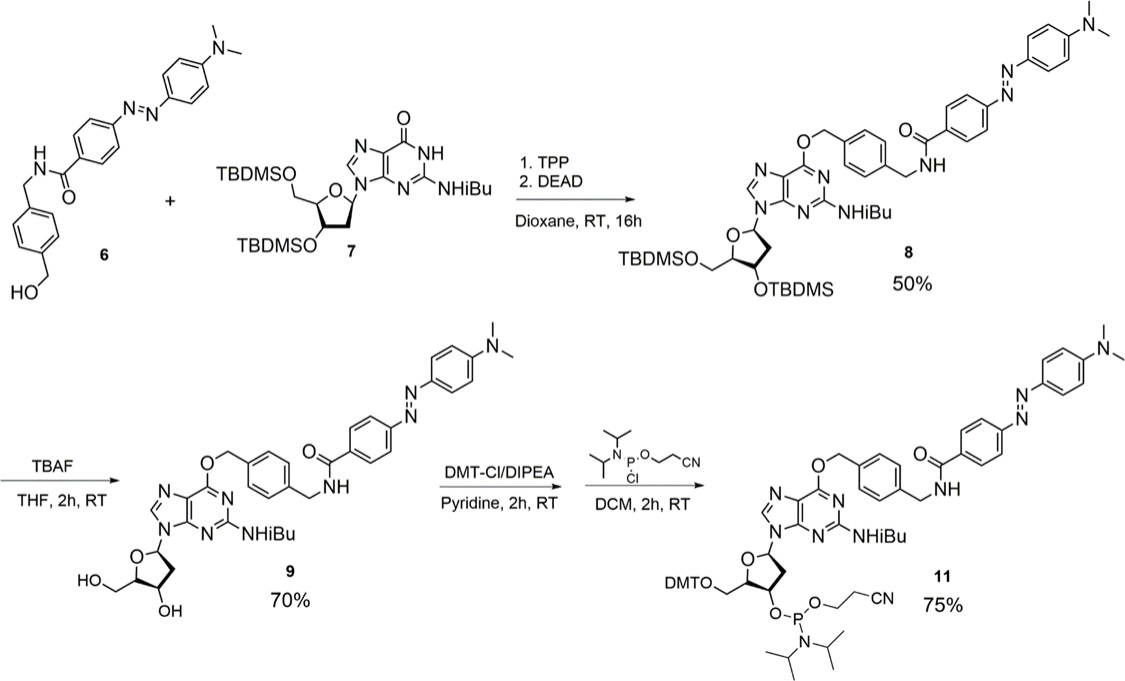
Synthesis of Dabcyl-BG nucleoside phosphoramidite 10, which was used as a monomer in preparation of chemosensors 1, 2, 3 and 4.

Our synthesis strategy (Fig 2) relied on modifying the BG nucleoside such that the final building block can be simply incorporated into oligonucleotides by solid phase DNA synthesis. The DABCYL quencher was chosen since it can efficiently quench nearby fluorophores by FRET and/or static quenching mechanisms [20]. The first half of the synthesis involved reacting commercially available (4-aminomethyl-phenyl)-methanol with NHS-ester DABCYL to generate compound **6** in high yields (see Supporting Information (SI) text for details). The second half entailed fully protecting 2’-deoxyguanosine by TBDMS and isobutyryl protection to provide compound **7**. Mitsunobu coupling of **6** and **7** produced quencher-substituted nucleoside **8** in 50% yield. Finally, deprotection of TBDMS followed by standard 5’-OH tritylation and 3’-OH phosphoramidite derivatization yielded the final monomer for DNA synthesis (**11**, Fig 2).

Incorporating **11** during DNA synthesis, we constructed several candidate MGMT probes to act as potential substrates for the enzyme. Probe designs were based on multiple concepts: first, MGMT has been shown to have no preference for repairing damage in ssDNA versus dsDNA [18], thus we designed the molecules to be active in single-stranded form. Second, MGMT has been reported to repair BG efficiently within short oligonucleotides greater than trimer in length [21], and so our candidate compounds were prepared in 4–5 nt lengths. Third, we envisaged applications in vitro and in cell lysates, and so modifications to enhance cell permeability were not carried out, since the modification might also be deleterious to enzyme activity. Lastly, MGMT displays no known selectivity in the DNA sequence for its repair activity, nor any strong preference for the positioning of the BG site within the DNA, providing the alkylated nucleoside is not incorporated directly at the 3’ end [22]. Thus our probe designs contained the monomer **11** at internal positions.

To complete the probe design, we chose a series of fluorophores that could be quenched efficiently by the nearby DABCYL-BG group. As one dye, perylene deoxynucleoside [23] was chosen since its cyan emission overlaps well with the absorption spectrum of DABCYL. In that case we would expect FRET-based quenching to occur by DABCYL-BG prior to MGMT repair. We also tested a series of longer-wavelength dye monomers, including FAM-dT, TAMRA-dT and Cy3 dyes, all of which can be quenched by DABCYL when placed in close proximity [20]. After standard oligonucleotide synthesis, probes were purified by HPLC and characterized by mass spectrometry (Table A in S1 File). Table 1 summarizes the MGMT probes synthesized along with their photophysical properties.

**Table 1:** Sequences and optical properties of chemosensors.

Chemosensor	Sequence ^a^	Ex. (nm)	Em. (nm)	Fold-change
1	A-dT^FAM^–G^DAB^-A	496	520	55 ± 5.0
2	Cy3-G^DAB^-A-A-A	550	560	30 ± 2.0
3	A-dT^TMR^-G^DAB^-A	565	580	12 ± 1.2
4	A-E-G^DAB^-A	454	475	50 ± 2.0

^a^ G^DAB^ denotes the Dabcyl-BG nucleoside

### Evaluation of Probes in Vitro

**Fig 3:**
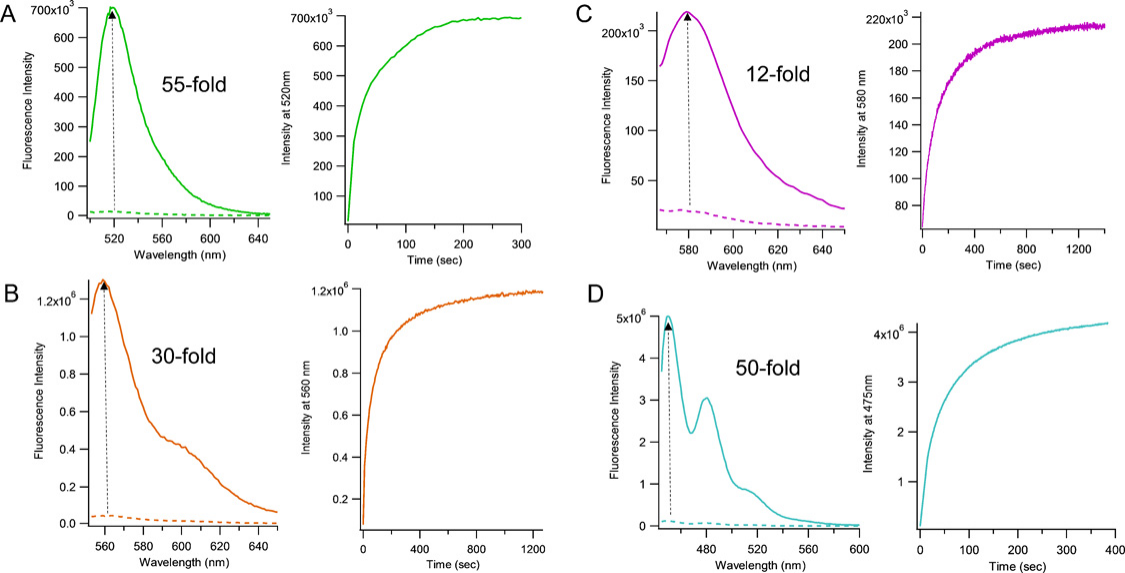
Performance of modified DNA chemosensors containing the Dabcyl-BG nucleoside. Fluorescence spectra showing the overall fold-changes in intensity observed when comparing fluorescence measured before (dashed line) and after (solid line) addition of purified MGMT protein are shown at left of each figure. Time courses (on the right) show time-dependent fluorescence increases immediately after addition of enzyme. Final probe and MGMT concentrations were 100 nM. Assays were run at 37°C in 70 mM HEPES buffer pH 7.8 containing 5 mM EDTA, 1 mM dithiothreitol and 50 μg/ml BSA. (A) chemosensor **1** containing dT^FAM^, (B), chemosensor **2** containing Cy3, (C), chemosensor **3** containing dT^TMR^ and (D), chemosensor **4** containing perylene nucleoside. Measurements were repeated 3 times. Standard deviations are provided in Table 1.

To test whether the probes could act as MGMT substrates and produce fluorescence responses upon repair, *in vitro* assays were carried out with purified human recombinant enzyme. Probes were incubated in a 1:1 stoichiometric ratio with MGMT (100 nM, 37°C) and fluorescence was monitored over time. Signals were found to increase for all probes, with maximum fluorescence reached in ca. 3–10 min (Fig 3). To confirm that reaction of the probe with MGMT was the origin of the fluorescent signal, crude reaction mixtures were analyzed by MALDI-MS. The data clearly showed the appearance of fully repaired oligonucleotides containing native guanine and lacking the DABCYL-benzyl moiety (Figure A in S1 File). This confirms that MGMT can recognize a quencher-modified BG and repair the lesion by transferring the O6 group from the substrate.

Quantitative fluorescence measurements allowed us to evaluate the signaling performance of these probes. The data revealed that the largest increase in signal upon reaction occurred with chemosensor **1** (FAM-substituted), resulting in a 55-fold change in green fluorescence after MGMT repair (Table 1). The reaction with this probe also occurred the fastest of the three probes, with complete repair achieved in ~3 min. We measured a K_m_ of 58 ± 17 nM (Figure B in S1 File), which is in the reported range for naturally substituted oligonucleotides containing BG (ED_50_ = 10–90 nM)[21]. Our measured value of k_cat_ (0.011 s^-1^) is moderately lower than the reported values for native *O*^6^-MedG (0.035–0.39 s^-1^) [24]. Thus we conclude that the multiple synthetic modifications in the probe have relatively little adverse effect on its ability to act as a substrate and reporter.

### Evaluating MGMT Inhibitors

**Fig 4:**
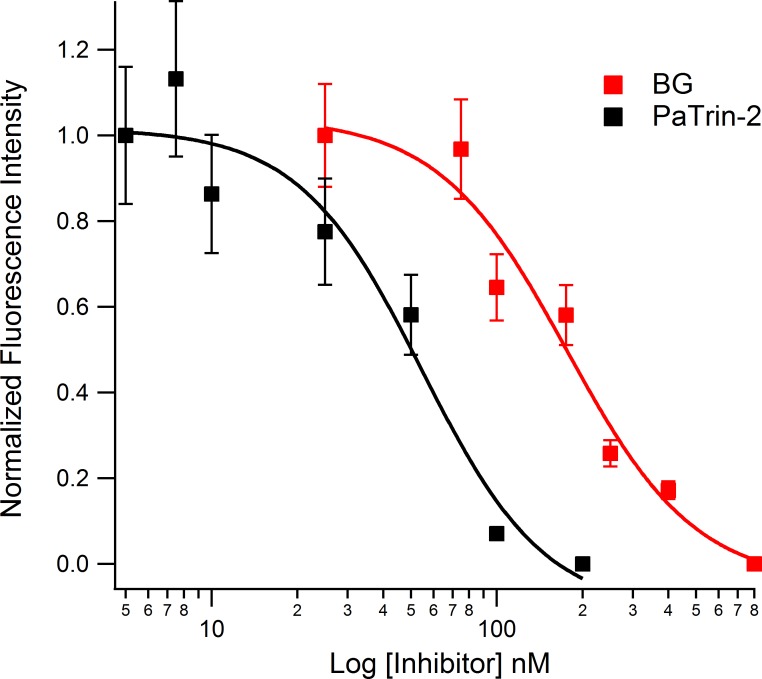
Evaluation of MGMT inhibitors with chemosensor 1. Incubation of purified MGMT enzyme with the inhibitors BG and PaTrin-2 led to a concentration dependent decrease in observed final fluorescence intensity, indicative of MGMT inhibition. MGMT (10 nM) was incubated with inhibitor for 10 min at 37°C in 70 mM HEPES buffer (pH 7.8) containing 5 mM EDTA, 1 mM dithiothreitol and 50 μg/ml BSA. Final fluorescence was acquired 10 min after addition of probe (10 nM). Data were normalized to measurements without inhibitor. Each data point is the average of 3 measurements.

We assessed fluorescein probe **1** for utility in testing inhibitors because of its efficient photophysical and biochemical performance relative to the other probes. Inhibitors of MGMT are currently being employed in clinical trials to improve the efficacy of temozolomide [7]. A simple assay to monitor MGMT activity would allow for direct evaluation of current inhibitors and/or the discovery of new inhibitors with different pharmacologic properties. To test the effectiveness of probe **1** in this application, we first employed the known inhibitor BG (free nucleobase), which inhibits MGMT by acting as a pseudosubstrate. BG is a moderately potent inhibitor with a reported IC_50_ of 200 nM [25]. MGMT was incubated first with 25–800 nM of BG at 37°C. After 10 min, an equimolar amount of probe was added and fluorescence was acquired after signals no longer changed. In comparison to the positive control (no inhibitor), the overall fluorescence change decreased with increasing concentrations of BG, with a measured IC_50_ = 176 ± 46 nM (Fig 4 and Figure C in S1 File). Next we employed the inhibitor PaTrin-2; this second compound also acts as a pseudosubstrate for MGMT but is considerably more potent than BG [25]. Pre-incubation of MGMT with 5–200 nM PaTrin-2, a lower concentration range, led to decreases in fluorescence with a measured IC_50_ = 54 ± 18 nM. The data establish that probe **1** can be used to evaluate inhibitors and measure their relative potencies.

### Differentiating MGMT Activity in Cell Lysates

Current methods for measuring MGMT activity in cellular samples require several steps involving lengthy workups [26]. Hence, we next tested whether FAM probe **1** could simplify the assay of MGMT activity in such samples. We first tested the selectivity of our probe for MGMT by measuring fluorescence in mammalian whole-cell extracts. To reduce possible background signals generated by nuclease activity, the probe was modified to contain 2’-OMe groups and phosphorothioate linkages. The combination of both modifications have been shown to increase resistance of oligonucleotides to nuclease activity [27]. In vitro tests confirmed that this nuclease-resistant probe (NR-**1**) behaved similarly to the unmodified probe **1** (Figure D in S1 File).

**Fig 5:**
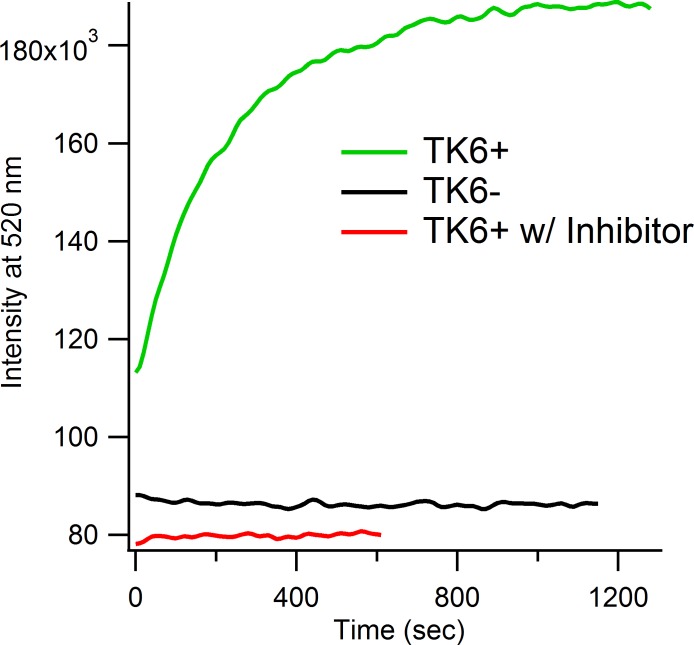
Selectivity for sensing MGMT activity. When chemosensor NR-**1** was added to TK6+ cell lysates (high amounts of MGMT) an immediate increase in fluorescence was observed. Addition of probe to TK6- cell lysates (MGMT knockout) or pretreating TK6+ with 2.5 μM inhibitor PaTrin-2 (10 min, 37°C) led to no change in fluorescence with time. Final probe concentration was 50 nM and total protein used was 200 μg. Data was acquired at 37°C. Measurements were repeated 3 times. Standard deviations are provided in Figure G in S1 File.

The probe NR-**1** was first tested in TK6+MGMT (TK6+) lymphoblastoid cell lysates, which had been previously complemented with an MGMT expression cassette to increase MGMT activity from otherwise undetectable levels [28]. Incubation with 50 nM probe resulted in an immediate increase in fluorescence with signals plateauing after 20 min at 37°C (Fig 5). By using the overall fluorescence fold-change, the amount of active MGMT was determined to be 2.9 pmol/mg (see Materials and Methods and Figure E in S1 File for calculation). To test whether this change is a result of MGMT repair, we pretreated TK6+ lysates with 2.5 μM of the potent MGMT inhibitor PaTrin-2. In this case, the NR-**1** probe gave no significant changes in fluorescence (Fig 5 and Figure F in S1 File). We then generated lysates from a TK6- cell line, in which promoter hypermethylation prevents expression of the native *MGMT* gene. Again, no detectable changes in fluorescein fluorescence were observed (Fig 5 and Figure F in S1 File). Thus, these experiments demonstrate that the NR**-1** probe is highly selective for the MGMT enzyme, and that changes in MGMT activity in cell lysates can be readily measured in a few minutes in a fluorescence cuvette.

**Fig 6:**
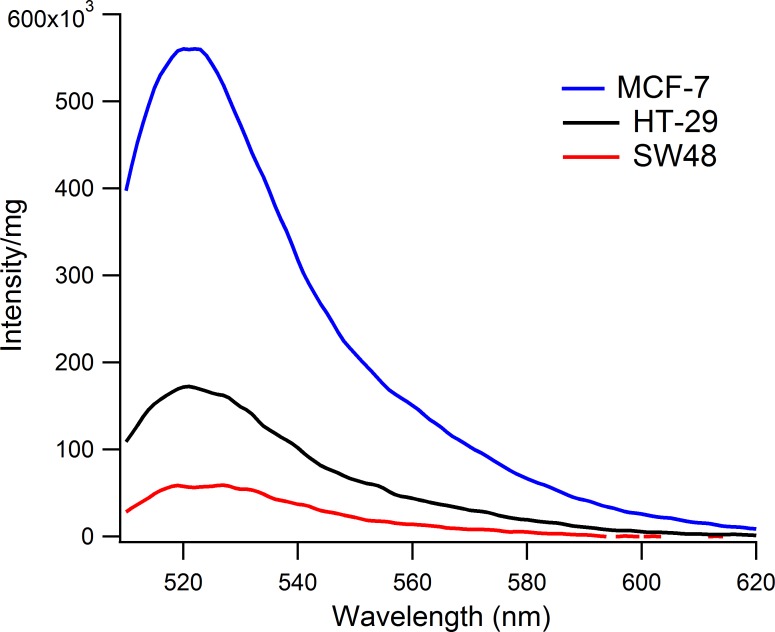
Differential detection of MGMT activity in tumor cell lysates. Chemosensor NR-**1** was added to whole cell lysates generated from MCF-7, HT-29 and SW48 cell lines. Final spectra were acquired after a plateau in fluorescence was observed. Spectra were then subtracted from background probe fluorescence (probe alone spectrum) and normalized by total amount of protein in order to compare activity levels. Final probe concentration was 50 nM. Data were acquired at 37°C. Measurements were repeated 3 times. Standard deviations are provided in Figure G in S1 File.

Next we tested whether probe NR-**1** can be used to differentiate levels of MGMT activity in different tumor cell lysates. We chose the breast cancer cell line MCF-7, which is known to have high levels of MGMT (3.24 pmol/mg of protein) [29], and two colon cancer cell lines, HT-29 (1.14 pmol/mg of protein) and SW48 (<0.05 pmol/mg of protein), known to have lower levels of activity [29,30]. We generated whole cell lysates and measured fluorescence time courses after addition of 50 nM probe. Once a steady signal was reached (40 min), spectra were acquired and corrected for background probe fluorescence and total protein content (see Methods). The resulting fluorescence levels would be indicative of MGMT activity, and in a relative sense, activity from different lysates could be compared (Fig 6). From the three cell lines we observe the highest MGMT activity from MCF-7 (2.6 ± 0.20 pmol/mg), followed by HT-29 (1.4 ± 0.35 pmol/mg) and the lowest from SW48 (0.61 ± 0.17 pmol/mg) (Fig 6). This trend is in good general agreement with previously reported levels of MGMT in these cell lines [29,30].

## Discussion

Our experiments show that probe NR-**1** enables exceedingly simple real-time measurement of MGMT-mediated repair by fluorescence, and thus offers a method that is much simpler, faster and more directly relevant to repair activity than current methods. It is well established that high MGMT activity in tumor cells confers resistance to alkylating agents [5]. As a result, the MGMT status within tumors has become a clinically important predictor of a patient’s response to chemotherapeutic drugs. Because of the relative difficulty in measuring protein activity directly by prior methods, the current method of choice for determining MGMT activity levels is measuring promoter CpG methylation for the *MGMT* gene [9]. Although correlations have been found, several studies have shown discrepancies between DNA methylation and MGMT levels [10]. For example, Kreth *et al*. [31] measured *MGMT* mRNA expression levels and found that 25% of glioblastoma samples showed an inverse correlation with promoter methylation. Lalezari *et al*. [32] also observed an inverse correlation using immunohistochemistry staining compared to promoter methylation in 44% of patient samples. These studies implicate other possible mechanisms for regulation beyond the level of transcription and as a consequence, the correlation between promoter CpG methylation and MGMT activity breaks down in a substantial number of patients. Therefore, measuring activity directly, as NR-**1** does, may provide a more clinically relevant assessment of MGMT [9,33]. The current trend toward increasing adoption of fluorescence assays in clinical applications [34,35] also suggests utility for the new probes.

To date, only a few reports of probes for MGMT activity have been described. Damoiseaux *et al*. [36] reported an oligonucleotide containing BG synthetically modified with biotin. The biotin moiety is transferred to the active site of MGMT and is sequentially immobilized on streptavidin-coated wells and detected by immunofluorescence staining. Although the method selects for active enzyme, subsequent reading out of activity still requires multiple steps. In addition, the probe design has not been tested with cell lysates. A report of a fluorescent probe was described by Li *et al*. [37] wherein BG was modified with a fluorophore and used in the free-base form to label active MGMT. However, the molecular design lacks a mechanism for fluorescence change upon labeling. Thus it would require development of a method to separate the reacted probe and enzyme from one another prior to fluorescence measurement, complicating its use and prohibiting its application in real-time assays for MGMT activity. During the completion of this manuscript, Yu *et al*. [38] reported BG modified with a fluorescent molecular rotor that increased in fluorescence upon labeling MGMT. However, fluorophore-labeled BG (or SNAP-tag probes) are poor substrates for wild-type MGMT [19,39] and as a consequence the fluorescence response with enzyme was slow, requiring high concentrations of probe (5 μM) to produce signals on a timescale of hours.

In contrast to the above approaches, NR-**1** and the other current probes are built from a DNA scaffold, the enzyme’s native target, which allows for the incorporation of fluorophores/quenchers to assay repair directly, and allows them to retain the superior activity of DNA substrates. Since the enzyme tolerates modifications on the benzyl substrate, the incorporation of a quencher group enables large fluorescence responses to occur upon DNA repair. Current practice measures MGMT from lysates or fixed cells generated from homogenized biopsied tumor tissue [9,33]. The current data show that NR-**1** can measure MGMT directly from lysates from multiple tumor cell lines in a timescale of minutes with only 50 nM probe (Fig 6). The single-step/direct design of probe NR-**1** may in principle provide rapid and quantitative measurements of MGMT activity in clinical samples from solid tumors.

In addition to solid tumors, a recent clinical study showed substantial promise for the use of temozolomide in the treatment of acute myeloid leukemia [8]. Patients were selected by their levels of MGMT; the authors employed a Western blot method to measure MGMT protein levels. Significantly, they reported difficulties in enrolling patients due to the slow nature of the assay, which required one week to obtain results. In contrast, the current chemosensor design offers a one-step optical measurement of protein activity from cells, yielding full quantitative responses in a few minutes.

Inhibiting MGMT with pseudosubstrates such as BG has been found to increase tumor sensitivity to alkylating agents [7]. Co-administering inhibitors with alkylating drugs has therefore become a clinically investigated strategy for cancer treatment [7]. Using the current chemosensor design, we were able to quantify inhibitory activity of two clinically approved MGMT inhibitors (Fig 4). The measured potency of these inhibitors was in good correlation with previous reports [18,25,40]. Furthermore, in light of the ongoing search for new MGMT inhibitors [41–43], chemosensors such as the current ones could be readily employed for high-throughput screening to identify new lead structures.

Lastly, it is known that polymorphisms in the *MGMT* gene can generate protein mutants that are resistant to free BG but still retain activity on BG-DNA [1,21,44]. Studies have shown that resistant forms may be acquired during BG or alkylating agent exposure [45–47]. It seems possible that DNA-based fluorogenic probes such as NR-**1** may be capable of characterizing activity from such MGMT mutants, and thus could be used to monitor this class of mutational resistance acquired during the course of therapy.

## Materials and Methods

### DNA synthesis

dT^FAM^, dT^TMR^, and Cy3 phosphoramidites were purchased from Glen Research. All oligonucleotides were synthesized on a model 394 DNA/RNA synthesizer (Applied Biosystems) with a DMT-off program. Fluorescent monomer E was prepared as described [48]. G^DAB^ phosphoramidite (compound **11**) was dissolved in anhydrous acetonitrile:dichloromethane (50:50) for DNA synthesis. The coupling of each monomer used standard 3′-to-5′ cyanoethyl phosphoramidite chemistry with 999 s coupling times for dT^FAM^, dT^TMR^, Cy3, G^Dab^ and E. Average coupling yields for the newly synthesized G^DAB^ was 95%. All chemosensors were synthesized on 1 μmol 3’PO_3_ CPG columns except for NR-**1**, which was made on a dA-CPG column. The nuclease-resistant chemosensor NR-**1** was synthesized using 2’-O-methyladenosine phosphoramidite and Sulfurizing Reagent II (Glen Research) with a 300s sulfurization time. Chemosensors **1**, **2**, **4** and NR-**1** were deprotected using 10% 1,8-Diazabicycloundec-7-ene (DBU) in anhydrous methanol for 10 days at room temperature to ensure complete removal of the guanine isobutyryl group. The mixtures were then dried by SpeedVac. Chemosensor **3** containing dT-TMR was deprotected in potassium carbonate (0.05 M) in methanol for 10 days, neutralized with glacial acetic acid, and then dried by SpeedVac. Complete removal of the isobutyryl group was not observed (~25%), but the product could be efficiently separated by HPLC purification.

### Purification of Chemosensors

All oligonucleotides were purified by HPLC on semi-preparative C18 column (length = 250 mm, ID = 10.0 mm) using triethylammonium acetate (TEAA) buffer (0.1M, pH 7.0) and acetonitrile. The following gradient was used: 5–95% acetonitrile at 1.5 ml/min flow in 35 min. The collected products were dried by SpeedVac, resuspended in water and stock solutions stored frozen at -20°C. The chemosensors were identified by MALDI-TOF (Table A in S1 File). The concentration of chemosensor **1** was measured in phosphate saline buffer pH 7.0, using the sum of the extinction coefficients for dabcyl and fluorescein at 500 nm (95 600 M^-1^cm^-1^). The concentrations of chemosensor **2** and **3** were measured in acetonitrile using the extinction coefficients at 550 nm (135 000 M^-1^cm^-1^) and 565 nm (89 000 M^-1^cm^-1^), respectively. The concentration of chemosensor **4** was measured in phosphate saline buffer pH 7.0 using the extinction coefficient of DABCYL at 480 nm (32 000 M^-1^cm^-1^). All chemosensors were stored at -20°C and were stable for at least 6 months.

### Fluorescence-based MGMT Assays

Purified recombinant human MGMT (His-tagged, expressed in *E*.*Coli*) was purchased from Creative BioMart. All assays were carried out at 37°C in 70 mM HEPES buffer (pH 7.8) containing 5 mM EDTA, 1 mM dithiothreitol and 50 μg/ml BSA. Fluorescence was performed in a quartz cuvette. Background fluorescence spectra were acquired after the probe (100 nM) had been incubated for 5 min at 37°C. The time course of fluorescence activation was recorded immediately after addition of MGMT (100 nM). Excitation and emission are listed in Table 1. Slit widths were 3 nm and 2 nm for excitation and emission, respectively. Final fluorescence spectra were acquired once a plateau in the fluorescence time course was observed.

For evaluating inhibitors, MGMT (10 nM or 100 nM) was preincubated with O^6^-benzylguanine (Santa Cruz Biotechnology) (25–800 nM) or PaTrin-2 (Cayman Chemical) (5–200 nM) for 10 min at 37°C in a 96-well plate. The final amount of DMSO did not exceed 0.5%. Fluorescence was recorded on a Thermo Fluoroskan Ascent FL fluorescent plate reader (Ex. 485 nm, Em. 538 nm). Chemosensor **1** (10 or 100 nM) was added and final fluorescence emission at 538 nm was acquired once a plateau was reached. All spectra were normalized to the spectrum obtained after the addition of probe to MGMT that had been preincubated for 10 min with 0.5% DMSO.

### Mammalian cell extracts

HT-29, SW48 and MCF-7 cell lines were purchased from ATCC. TK6+ and TK6- were provided by the Leona Samson lab (MIT). HT-29 and SW48 were grown in McCoys 5A modified medium supplemented with fetal bovine serum (10%), penicillin (100 U mL^-1^) and streptomycin (100 μg mL^-1^). TK6+ and TK6- were grown in DMEM supplemented with fetal bovine serum (15%), penicillin (100 U mL^-1^) and streptomycin (100 μg mL^-1^). All cell lines were cultured in a humidified incubator at 37°C with 5% CO_2_. Cell extracts were obtained by the protocol adapted from Folco *et al*. [49], with the following changes: the cells (~2 x 10^6^) were collected by trypsinization at 37°C with 5% CO_2_; Roche complete mini EDTA-free tablets were used for protease inhibition; the cells were lysed by passing 15 times through a 21 gauge needle and 10 times through a 16 gauge needle. The nuclear fraction was pooled with the cytosolic fraction. Extractions were partitioned in 100 μL aliquots and stored at -80°C until used. Final buffer was: 10 mM HEPES (pH 7.9), 1.5 mM MgCl_2_, 10 mM KCl, 0.5 mM dithiothreitol with 5% glycerol and 1x protease inhibitor. Total protein concentration was determined by a Bradford assay.

### Fluorescence Assays with Extracts

Fluorescence assays with cell extracts were performed in the buffer used during lysis (10 mM HEPESpH 7.9, 1.5 mM MgCl_2_, 10 mM KCl, 0.5 mM dithiothreitol, 5% glycerol, 1x protease inhibitor) plus the addition of 5 mM EDTA. Fluorescence was acquired with chemosensor NR-**1** alone (50 nM) and lysates alone to correct for background fluorescence. After addition of probe (50 nM) to lysates, fluorescence at 520 nm was acquired with time. Once a steady signal was reached (~40 min), final spectra was measured and subtracted from background spectra. The corrected spectra was then divided by the amount of total protein used in the assay. Any fluorescence signal observed on the final spectra represents MGMT activity.

### Calculation of MGMT Activity in Extracts

MGMT activity was calculated as follows: The final fluorescence fold-change by NR- **1** in cell lysates was determined using 200 μg of TK6- lysate with 50 nM probe and addition of various amounts of purified MGMT (6–75 nM), followed by incubation for 1 h at 37°C in a 60 μL cuvette to generate a calibration curve (Figure E in S1 File). The overall fold-change was then measured following incubation of 50 nM NR-**1** with 1 mg protein extract from TK6+, MCF-7, HT-29 or SW48 using the same assay conditions. Fold change in fluorescence was then divided by the total mg of lysates used in the assay. Using the resulting normalized fold change per mg lysate, the concentration of MGMT (pmol MGMT per mg lysate) was then calculated from the calibration curve. Note: the fold changes observed were all greater than 1.5-fold but less than 10-fold to remain within the linear range of the assay between 6 and 50 nM MGMT. For MCF-7, HT-29 and SW48, 1 mg of lysate was used to generate signal to stay within the linear range of the calibration curve. For TK6+, 200 μg of lysates were used to stay within the linear range of the calibration curve.

### Kinetic Parameters

Michaelis–Menten curve was generated by preincubating MGMT (25 nM) in 70 mM HEPES buffer (pH 7.8) containing 5 mM EDTA, 1 mM dithiothreitol and 50 μg/ml BSA at 37°C for 5 min. Chemosensor **1** (25–300 nM) was then added and the initial velocity was measured at 520 nm over the first 2 min. The curve was fit to the Hill equation (IGOR Pro) where n = 1 to yield the Michaelis-Menten equation. The K_m_ and V_max_ could then be determined. For k_cat_ (*k*_cat_ (*k*_cat_ = *V*_max_/[enzyme]), the fluorescence units with time (at saturation) was converted to product produced with time to obtain V_max_ (nM/s). Since MGMT is not a turnover enzyme, the maximum of product produced is equal to the concentration of enzyme used. Also note that “k_cat_” values are technically measurements of inactivation and not catalysis; thus “k_inact_” is more accurate [24].

## Supporting Information

S1 File Supporting Information for *Fluorogenic Real-time Reporters of DNA Repair by MGMT*, *a Clinical Predictor of Antitumor Drug Response*. (DOC)
