# Supplementary material for: Fluorogenic Real-Time Reporters of DNA Repair by MGMT, a Clinical Predictor of Antitumor Drug Response
Source: PLoS One. 2016 Apr 1;11(4):e0152684. doi: 10.1371/journal.pone.0152684 (PMC4818092; doi:10.1371/journal.pone.0152684)
Supplement: S1 File — (DOC) [file pone.0152684.s001.doc]

**Supporting Information**

**Fluorogenic Real-time Reporters of DNA Repair by MGMT, a Clinical Predictor of Antitumor Drug Response**

Andrew A. Beharry, ^1^ Zachary D. Nagel, ^2^ Leona D. Samson, ^2^ and Eric T. Kool^1^

^1^Department of Chemistry, Stanford University, Stanford, CA 94305-5080

^2^Departments of Biological Engineering and Biology, Massachusetts Institute of Technology, Cambridge, MA 02139

**General Information**. Chemicals and solvents were purchased from Aldrich unless otherwise stated. 1H-NMR and 13C-NMR were taken on Varian Innova 400-MHz and 500-MHz NMR spectrometer. Chemical shifts are reported in ppm downfield from tetramethylsilane (TMS). Data are reported as follows: chemical shift, multiplicity: singlet (s), doublet (d), multiplet (m), coupling constant (J) in Hz and integration. High-resolution mass spectrometry (HRMS) was performed by the Vincent Coates Foundation Mass Spectrometry Laboratory, Stanford University Mass Spectrometry. Semi-preparative high performance liquid chromatography was performed on a LC-CAD Shimadzu liquid chromatograph, equipped with a SPD-M10A VD diode array detector and a SCL 10A VP system controller. Oligo masses were determined by the Stanford University Protein and Nucleic Acid Facility. Absorbance spectra were obtained on a Cary 100 Bio UV-Vis spectrometer. Fluorescence emission spectra were obtained on a Jobin Yvon-Spex Fluorolog 3 spectrometer.

**Perylene (E) monomer synthesis**. The synthesis of E was carried out as previously described (Gao et al., 2002)

**
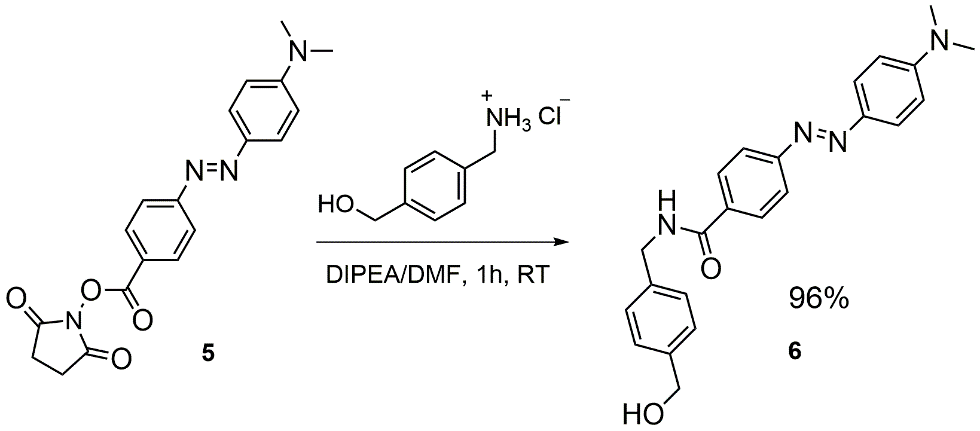
**

**(E)-4-((4-(dimethylamino)phenyl)diazenyl)-N-(4-(hydroxymethyl)benzyl)benzamide (6). 5** was synthesized as previously describe (Sun et al., 2007). **5** (1.08g, 2.9 mmol) was dissolved in dry dimethylformamide (25 ml). (4-(Aminomethyl)phenyl)methanol hydrochloride (Astatech, Inc.) (450 mg, 2.6 mmol) was then added, followed by slow addition of N,N-diisopropylethylamine (7.2 mmol, 1.25 ml). The reaction was stirred at room temperature for 1 h then concentrated under vacuum. The solid was purified by flash silica chromatography (EtOAc/hexanes 2:1 to 100% EtOAc) to yield the product as a bright orange powder (968 mg, 96%). ^1^H-NMR (400 MHz, DMSO-d_6_): δ = 9.12-9.15 (t, J=6.0, 1H), 8.00-8.02 (d, J=8.5, 2H), 7.79-7.82 (dd, J=8.8, 4H), 7.26 (d, J=2, 4H), 6.81-6.84 (d, J=9.3, 2H), 4.44-4.47 (m, 4H), 3.05 (s, 6H). ^13^C-NMR (500 MHz, DMSO-d_6_): δ = 165.63, 154.05, 152.87, 142.65, 141.08, 138.01, 134.62, 128.46, 127.11, 126.51, 125.17, 121.61, 111.61, 62.74, 42.57, 39.89. HRMS (m/z): [M+H]^+^ calculated for C_23_H_25_N_4_O_2_ 389.1977 Da, found 389.1972 Da.

**
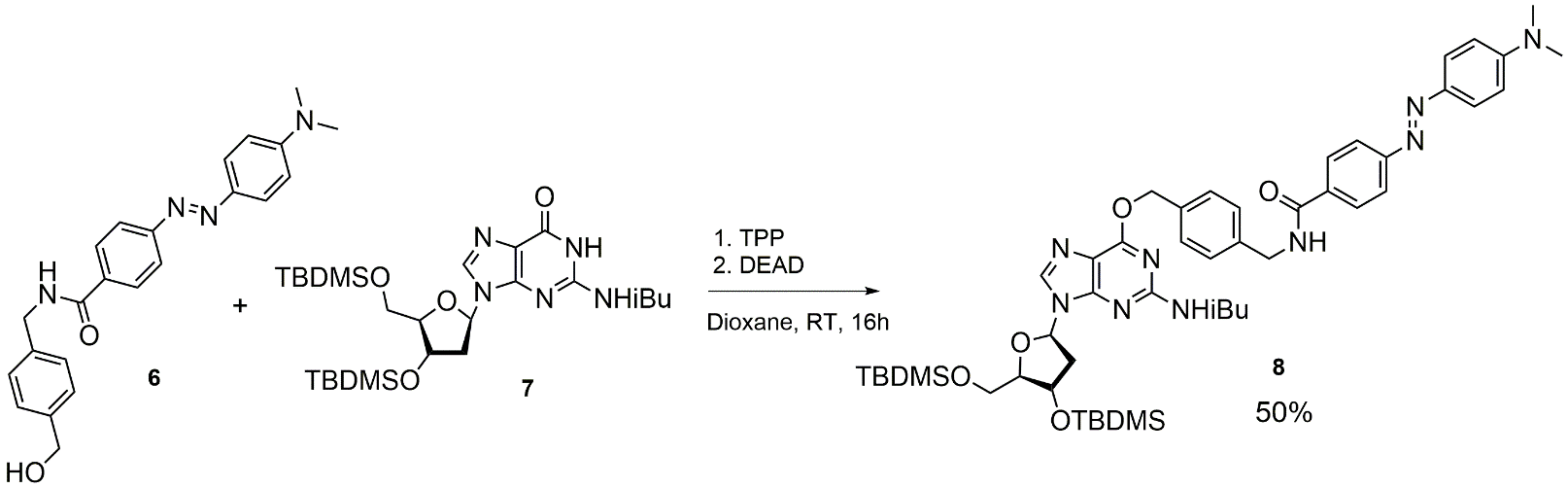
**

**(E)-N-(4-(((9-(4-((tert-butyldimethylsilyl)oxy)-5-(((tert-butyldimethylsilyl)oxy)methyl)tetrahydrofuran-2-yl)-2-isobutyramido-9H-purin-6-yl)oxy)methyl)benzyl)-4-((4-(dimethylamino)phenyl)diazenyl)benzamide (8). 7** was synthesized as previously described (Pon et al., 1986; Woo et al., 1993). **7** (0.17 mmol, 100 mg) was dissolved in dry dioxane (2 ml) to which triphenylphosphine (0.35 mmol, 91 mg) and **6** (0.35 mmol, 135 mg) was then added. The mixture was stirred for 30 min at room temperature under argon atmosphere. Diethyl azodicarboxylate (40% solution in toluene, 0.35 mmol, 152 µL) was mixed with dry dioxane (2 ml) then added dropwise to the reaction flask. The solution was stirred for 16 h at room temperature, then concentrated under vacuum. The compound was purified by flash silica chromatography (EtOAc/hexanes 1:1) to yield an orange powder/foam (80 mg, 50%). The compound was used without further purification for the next step. ^1^H-NMR (500 MHz, CDCl_3_): δ = 8.16 (s, 1H), 7.97-7.99 (d, J=8.5, 2H), 7.84-7.89 (dd, J= 8.3, J’= 9.0, 4H), 7.38-7.39 (d, J= 7.8, 2H), 7.27-7.29 (d, J=7.29, 2H), 6.73-6.75 (d, J =9.0, 2H), 6.39-6.41 (t, J=6.3, 1H), 5.51 (s, 2H), 4.62-4.63 (d, J=5.4, 2H), 4.52-4.55 (m, 1H), 3.84-3.87 (m, 1H), 3.75-3.79 (m, 1H), 3.46-3.50 (m, 1H), 3.09 (s, 6H), 2.55-2.60 (m, 1H), 2.38-2.43 (m, 1H), 2.26-2.31 (m, 1H), 1.26-1.28 (m, 6H), 0.90-0.92 (2s, 18H), 0.099-0.11 (2s, 12H). ^13^C-NMR (500 MHz, CDCl_3_): δ =179.17, 167.02, 160.44, 14.95, 152.73, 152.41, 151.87, 143.55, 139.98, 134.96, 134.26, 128.77, 128.09, 127.97, 125.38, 122.15, 118.18, 111.43, 87.94, 84.21, 71.84, 68.39, 62.80, 62.15, 43.80, 41.44, 40.31, 33.90, 2.96, 25.74, 24.96, 19.29, 18.41, 17.98, -4.67, -4.79, -5.37, -5.48. HRMS (m/z): [M+H]^+^ calculated for C_49_H_70_N_9_O_6_Si_2_ 936.4987 Da, found 936.4982 Da.

**
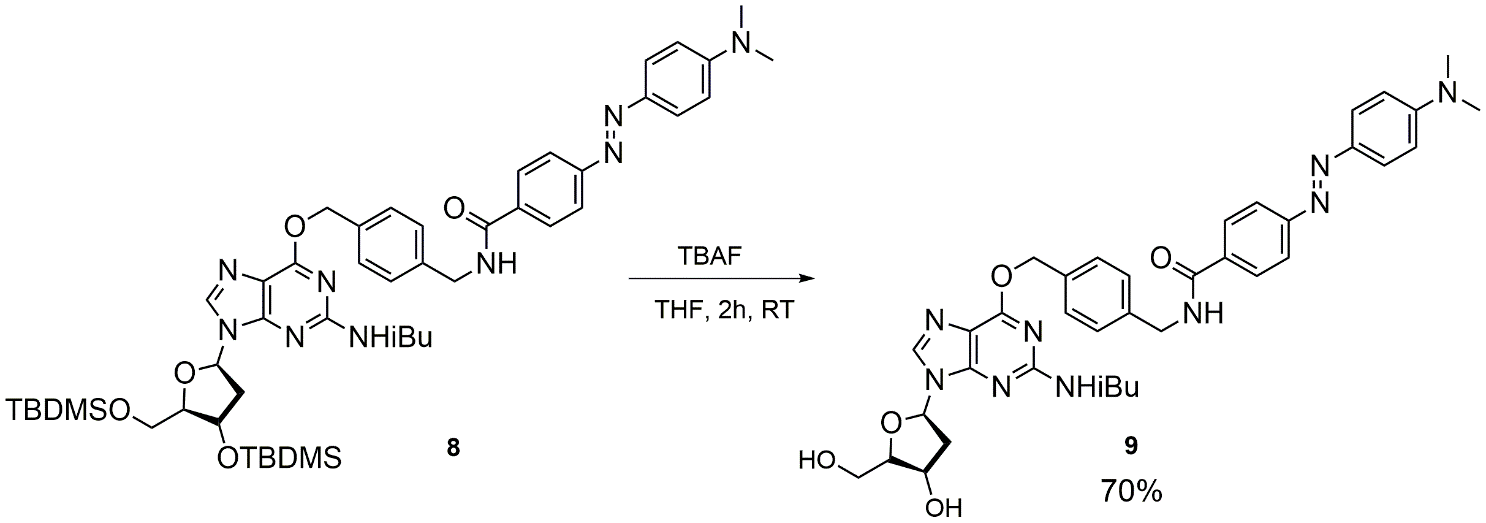
**

**(E)-4-((4-(dimethylamino)phenyl)diazenyl)-N-(4-(((9-(4-hydroxy-5-(hydroxymethyl)tetrahydrofuran-2-yl)-2-isobutyramido-9H-purin-6-yl)oxy)methyl)benzyl)benzamide (9). 8** (0.14 mmol, 130 mg) was dissolved in THF (4ml) to which tetra-n-butylammonium fluoride (1M in THF, 0.45 mmol, 450 µL) was added dropwise and stirred for 2 h at room temperature. The reaction was quenched with brine and the aqueous layer was extracted 3 times with EtOAc. The organic layer was combined, dried over sodium sulfate then concentrated under vacuum. The compound was purified by flash silica chromatography (EtOAc/Hexanes 1:1 then 100% CH_2_Cl_2_ to 10% MeOH/90% CH_2_Cl_2_) to yield an orange powder (69 mg, 70%).^1^H-NMR (400 MHz, DMSO-d_6_): δ = 10.42 (s, 1H), 9.14-9.18 (t, J = 5.9, 1H), 8.43 (s, 1H), 8.01-8.04 (d, J = 8.5, 2H), 7.80-7.83 (d, J= 8.8, 4H), 7.52-7.54 (d, J = 8.1, 2H), 7.34-7.37 (d, J = 8.1, 2H), 6.82-6.85 (d, J = 9.3, 2H), 6.29-6.34 (t, J=6.7, 1H), 5.59 (s, 2H), 5.31 (s, br, 1H), 4.90 (s, br, 1H), 4.48-4.50 (d, J=5.9, 2H), 4.40 (s, 1H), 3.83-3.84 (m, 1H), 3.52-3.56 (m, 2H), 3.06 (s, 6H), 2.84-2.88 (m, 1H), 2.63-2.69 (m, 1H), 2.22-2.30 (m, 1H), 1.07-1.10 (d, J= 6.8, 6H). ^13^C-NMR (500 MHz, DMSO-d_6_): δ = 175.09, 165.70, 159.71, 154.06, 152.88, 152.76, 152.04, 142.65, 141.12, 139.81, 134.77, 134.56, 129.01, 128.49, 127.45, 125.18, 121.61, 117.39, 111.62, 87.88, 83.21, 70.68, 67.58, 61.63, 57.53, 42.59, 34.49, 23.10 19.41. HRMS (m/z): [M+H]^+^ calculated for C_37_H_42_N_9_O_6_ 708.3257 Da, found 708.3253


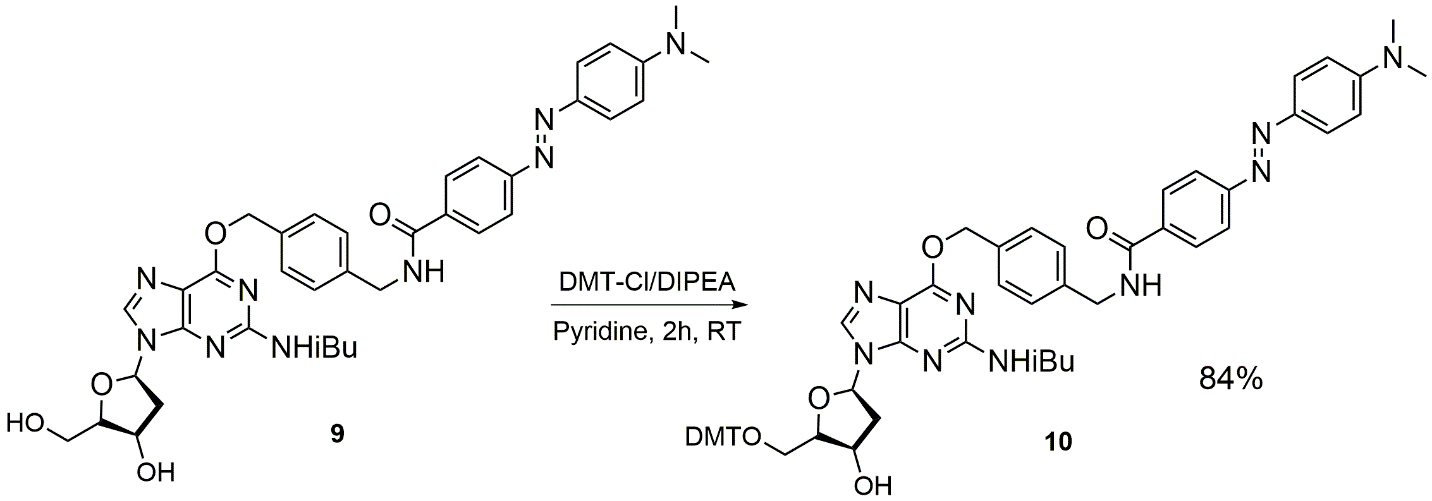


**(E)-N-(4-(((9-(5-((bis(4-methoxyphenyl)(phenyl)methoxy)methyl)-4-hydroxytetrahydrofuran-2-yl)-2-isobutyramido-9H-purin-6-yl)oxy)methyl)benzyl)-4-((4 (dimethylamino)phenyl)diazenyl)benzamide (10). 9** (0.20 mmol, 140 mg) was co-evaporated three times with dry pyridine (3 ml) and dissolved in pyridine (5.5 ml) to which N,N-diisopropylethylamine (1.3 mmol, 235 µl) was added. 4-4’-dimethoxytriphenylmethylchloride (0.59 mmol, 200 mg) was dissolved in 1.5 ml dry pyridine then added dropwise to the reaction flask. The reaction was stirred at room temperature for 1.5 h then quenched with MeOH (0.1 ml, stirred for 10 min) and concentrated under vacuum. The compound was purified by flash silica chromatography (EtOAc/hexanes 2:1 to 5% MeOH/3% trimethylamine in EtOAc to yield an orange foam (169 mg, 84%).

^1^H-NMR (500 MHz, CDCl_3_): δ = 8.19 (s, 1H), 7.89-7.94 (dd, J=9.0, J’= 8.3, 4H), 7.83-7.84 (d, J=8.3, 2H), 7.36-7.41 (dd, J=8.1, J’= 7.8, 4H), 7.21-7.30 (m, 9H), 6.75-6.78 (m, 6H), 6.52-6.55 (t, J=6.6, 1H), 5.47-5.53 (m, 2H), 4.72 (s, 1H), 4.58-4.59 (d, J=5.4, 2H), 4.19-4.22 (m, 1H), 3.74 (s, 6H), 3.34-3.42 (m, 2H), 3.11 (s, 6H), 2.78-2.85 (m, 1H), 2.67-2.72 (m, 1H), 2.54-2.59 (m, 1H), 1.18-1.21 (t, J=6.1, 6H). ^13^C-NMR (500 MHz, CDCl_3_): δ = 175.89, 166.91, 160.36, 158.40, 154.96, 152.72, 152.50, 151.68, 144.49, 143.52, 140.13, 138.23, 135.67, 135.9, 135.04, 134.13, 129.99, 128.64, 128.05, 127.97, 127.81, 126.84, 125.34, 122.12, 118.33, 113.08, 111.39, 95.50, 86.39, 84.09, 72.24, 68.24, 63.98, 55.14, 43.78, 40.68, 40.25, 34.45, 19.28. HRMS (m/z): [M+Na]^+^ calculated for C_58_H_59_N_9_NaO_8_ 1032.4384 Da, found 1032.4379 Da.


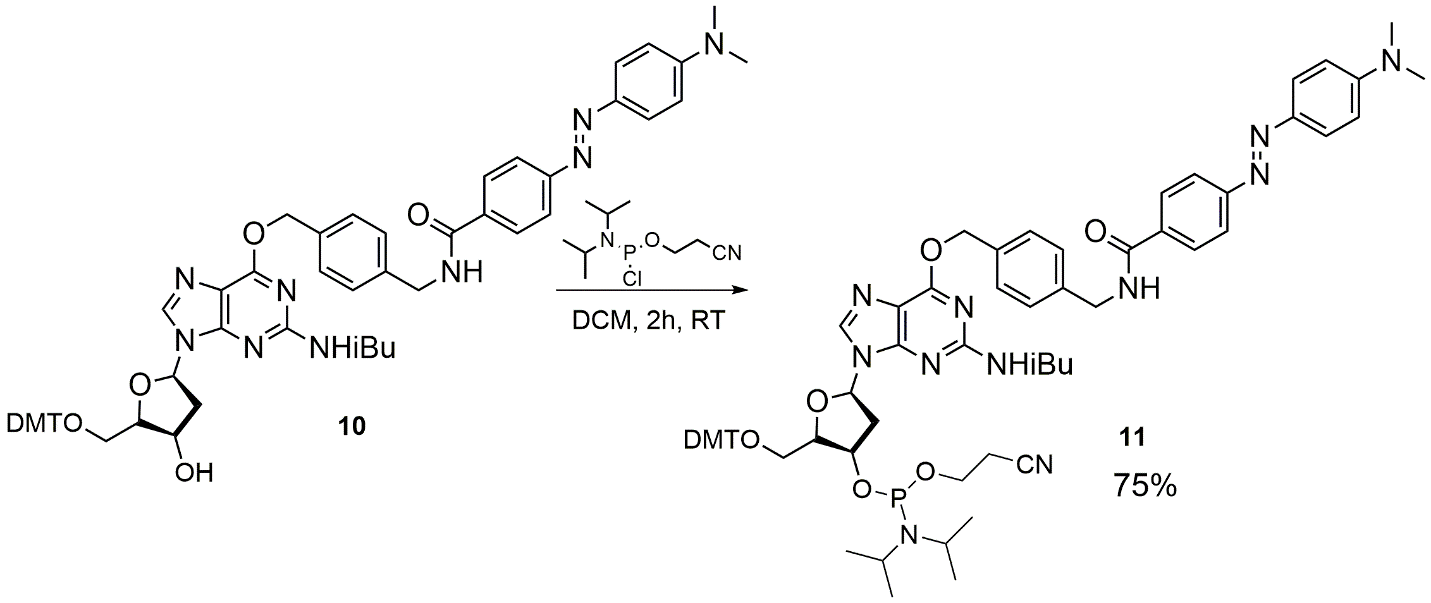


(**E)-2-((bis(4-methoxyphenyl)(phenyl)methoxy)methyl)-5-(6-((4-((4-((4-(dimethylamino)phenyl)diazenyl)benzamido)methyl)benzyl)oxy)-2-isobutyramido-9H-purin-9-yl)tetrahydrofuran-3-yl (2-cyanoethyl) diisopropylphosphoramidite** **(11)**. **10** were transformed to a 3’-O-phosphoramidite derivative and used immediately for solid-phase DNA synthesis. **10** (0.15 mmol, 150 mg) was dissolved in dry CH_2_Cl_2_ (3 ml) under argon atmosphere. Addition of DIPEA (0.90 mmol, 156 µL), then 2-cyanoethyl N,N-diisopropylchlorophosphoramidite (0.45 mmol, 100 µL) was added dropwise to the reaction flask. The reaction mixture was stirred at room temperature for 1 h and then concentrated under vacuum. The residue was purified by flash silica chromatography (EtOAc with 3% triethylamine) to yield an orange foam (136 mg, 75%). ^31^P-NMR (500 MHz, CDCl_3_): δ = 149.73, 149.71

**Table A. MALDI-TOF characterization of chemosensors**


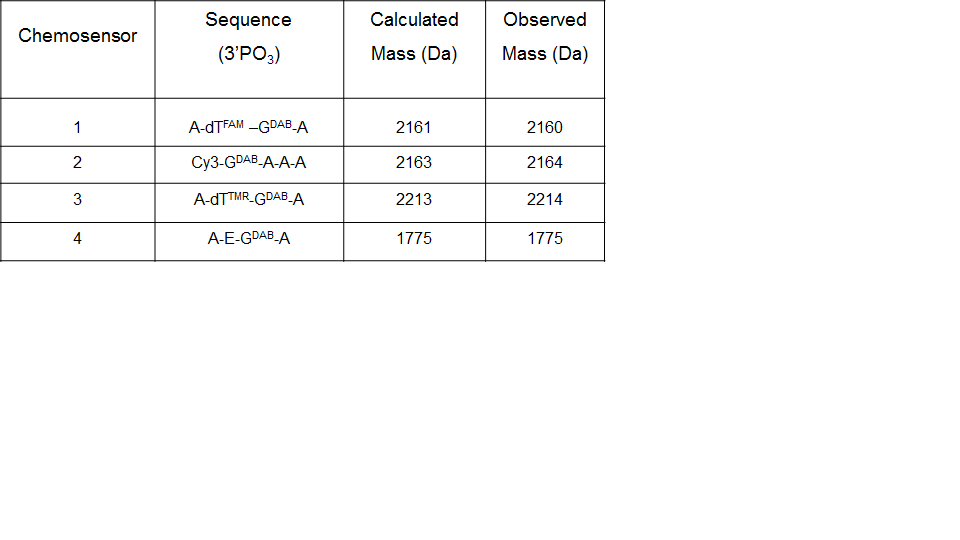


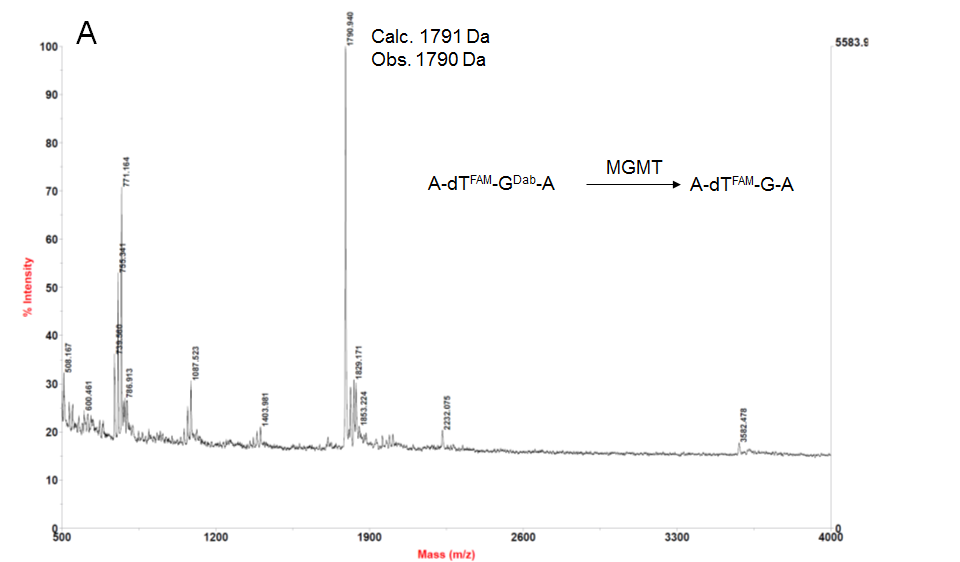


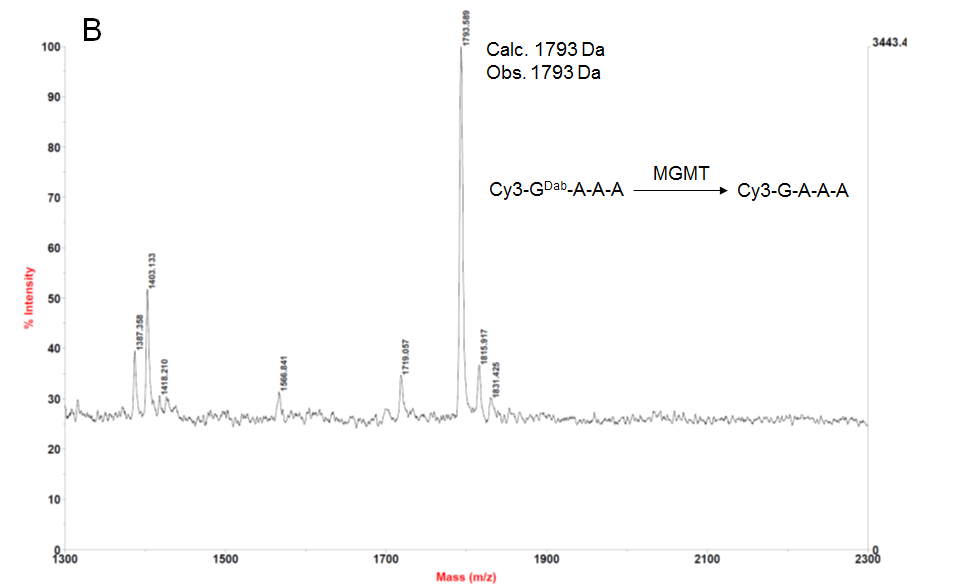


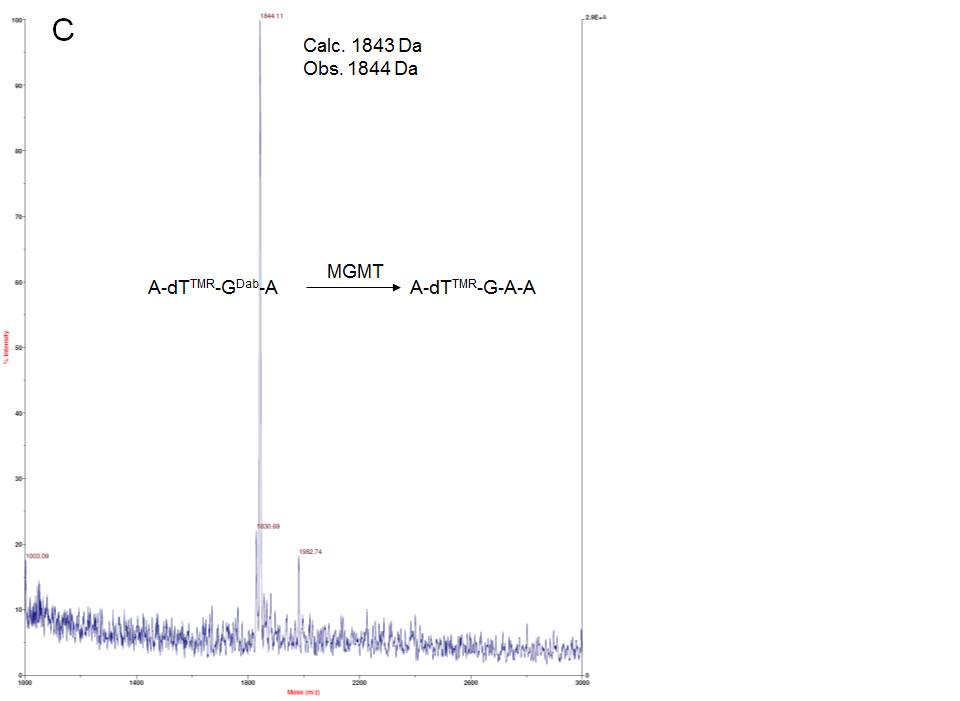


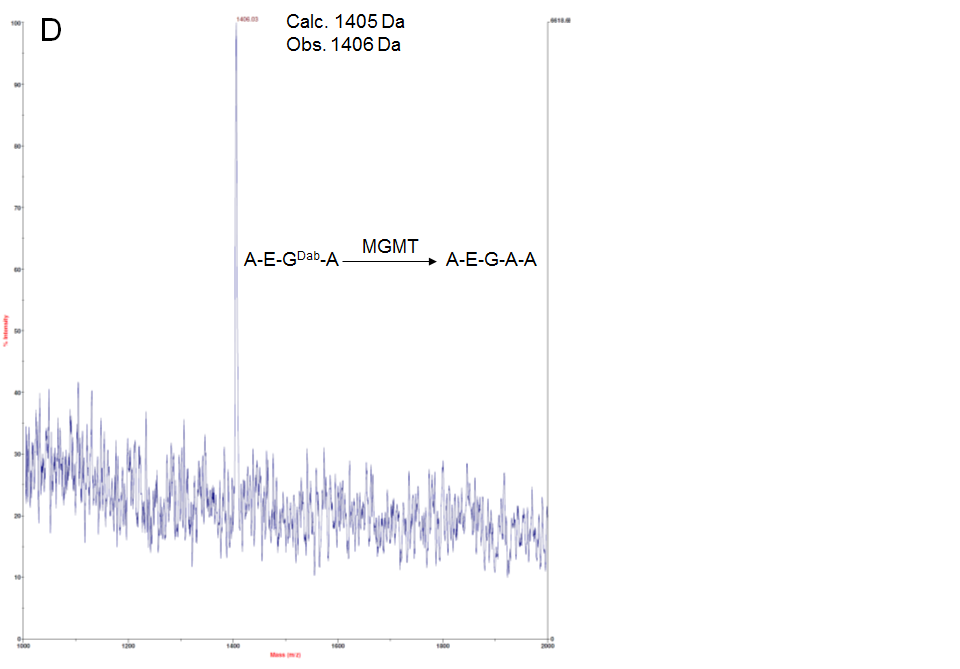


**Figure A**. **MALDI-TOF characterization of chemosensors after reaction with MGMT enzyme**. A, spectrum of chemosensor **1**, B, spectrum of chemosensor **2**, C, spectrum of chemosensor **3** and D, spectrum of chemosensor **4**.


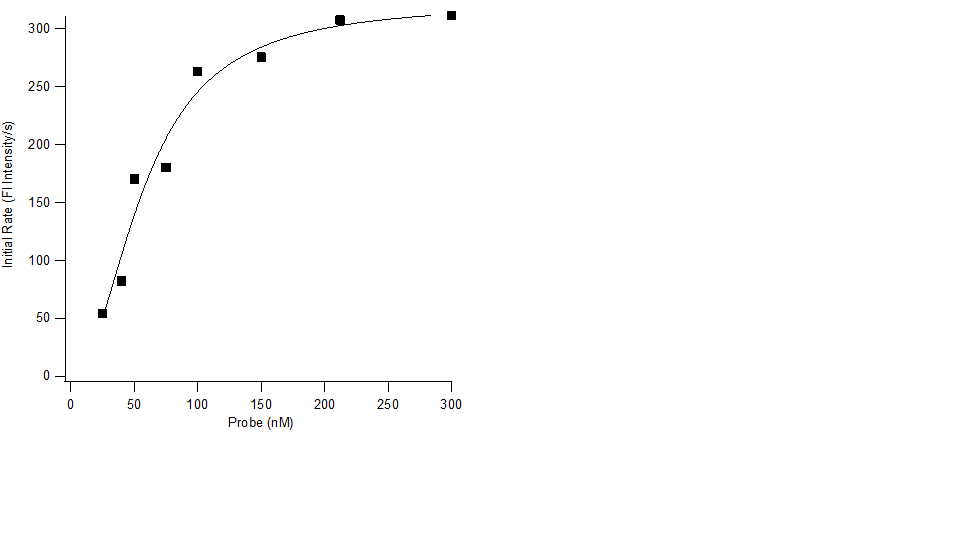


**Figure B**. **Michaelis–Menten curve of MGMT with chemosensor 1**. 25 nM MGMT was incubated with varying concentrations of chemosensor **1** in in 70 mM HEPES buffer (pH 7.8) containing 5 mM EDTA, 1 mM dithiothreitol and 50 µg/ml BSA at 37 °C. The curves were fitted to the Hill equation (n=1) with IGOR Pro software (**—**).


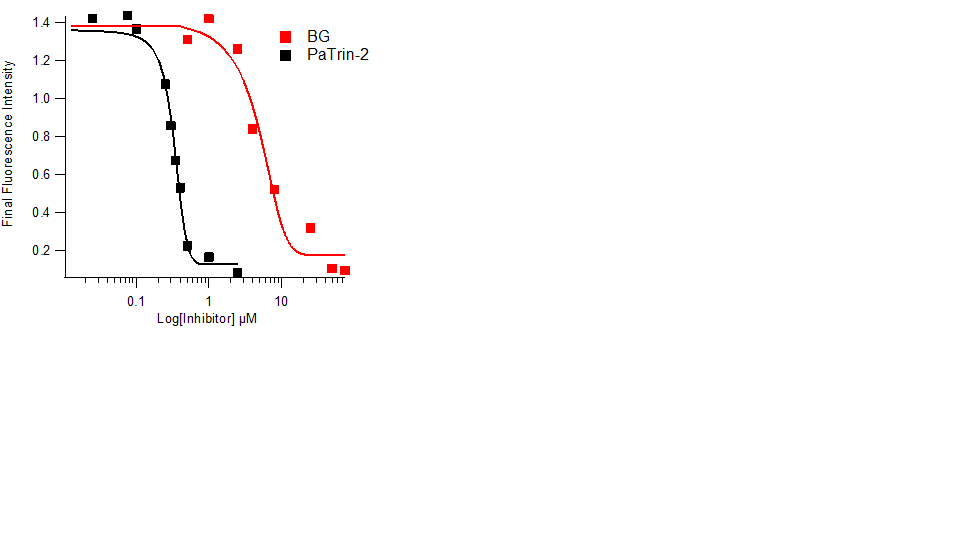


**Figure C**. **Evaluation of MGMT inhibitors with chemosensor** **1**. MGMT (100 nM) was incubated with inhibitor for 10 min at 37 °C in 70 mM HEPES buffer (pH 7.8) containing 5 mM EDTA, 1 mM dithiothreitol and 50 µg/ml BSA. Final fluorescence was acquired 10 min after addition of probe (100 nM). For BG, IC_50_ = 4.1 ± 0.54 µM. For PaTrin-2, IC_50_ = 0.32 ± 0.0077 µM.


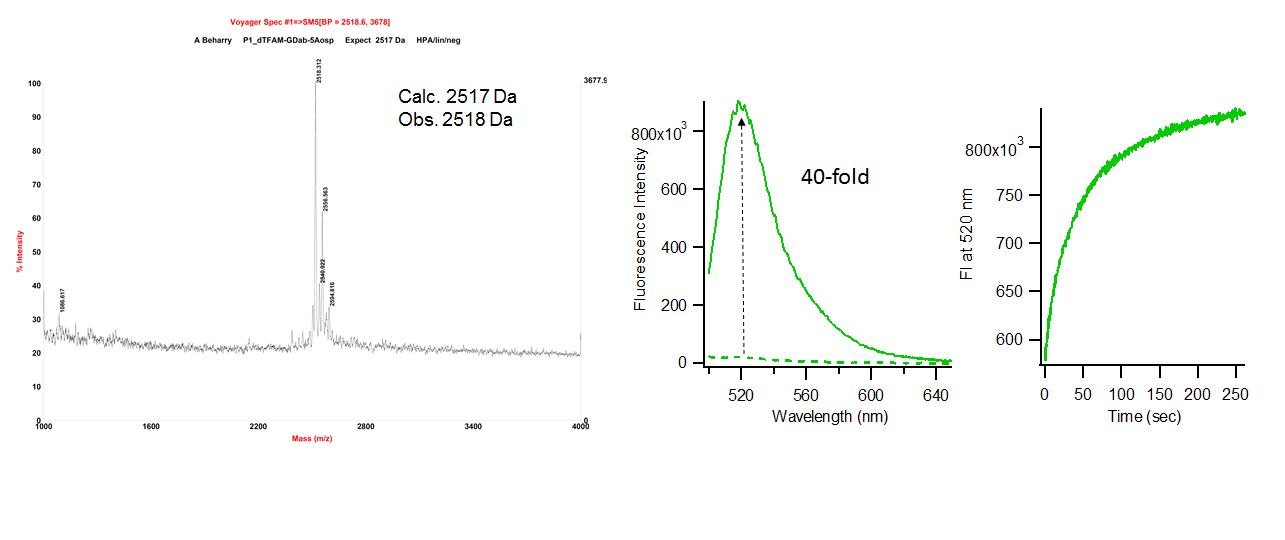


**Figure D**. **Characterization of NR-1**. Sequence: A_o_-ps-dT^FAM^-ps-G^Dab^-ps-A_o_-ps-A, where “A_o_” denotes adenosine containing 2’OMe groups and “ps” denotes phosphorothioate linkages. Left, MALDI-TOF of synthesized product. Right, fluorescence spectra and time course before and after addition of MGMT. Concentrations of probe and enzyme were 100 nM. Assay performed at 37° C in in 70 mM HEPES buffer (pH 7.8) containing 5 mM EDTA, 1 mM dithiothreitol and 50 µg/ml BSA.

**
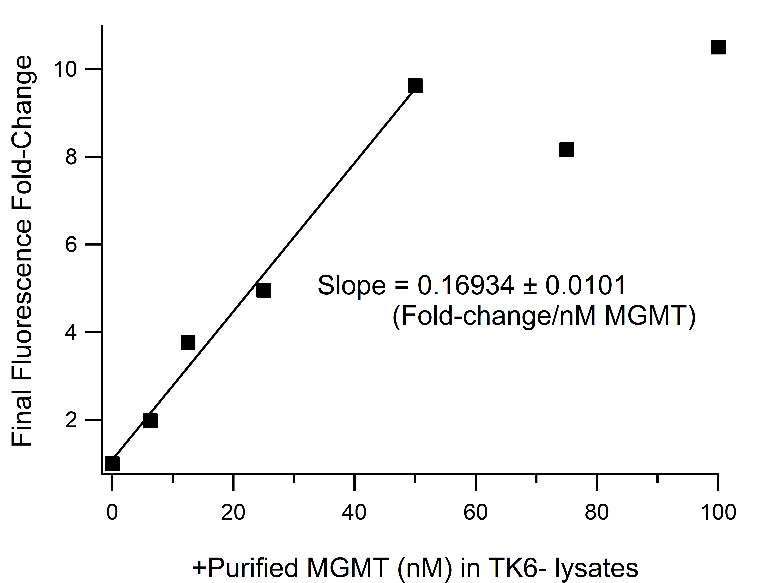
**

**Figure E**. **Calibration curve for MGMT activity calculation.** NR-1 (50 nM) incubated in TK6- (200 µg) cell lysates, followed by the addition of known amounts of purified MGMT. The final fluorescence fold change (after 30 min, 37 °C) was measured, plotted and the linear range of the assay established. Data falling in the linear range of the assay were used to generate a calibration curve.


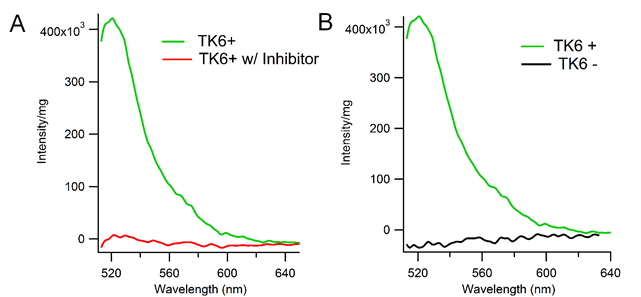


**Figure F. Selective MGMT sensing in TK6 cell lysates.** Full spectra obtained after a plateau in fluorescence signal change was observed. Spectra were subtracted from background probe fluorescence (probe alone spectrum) and normalized by total mg of protein. Final probe concentration was 50 nM and total protein used was 200 µg. Data was acquired at 37 °C.


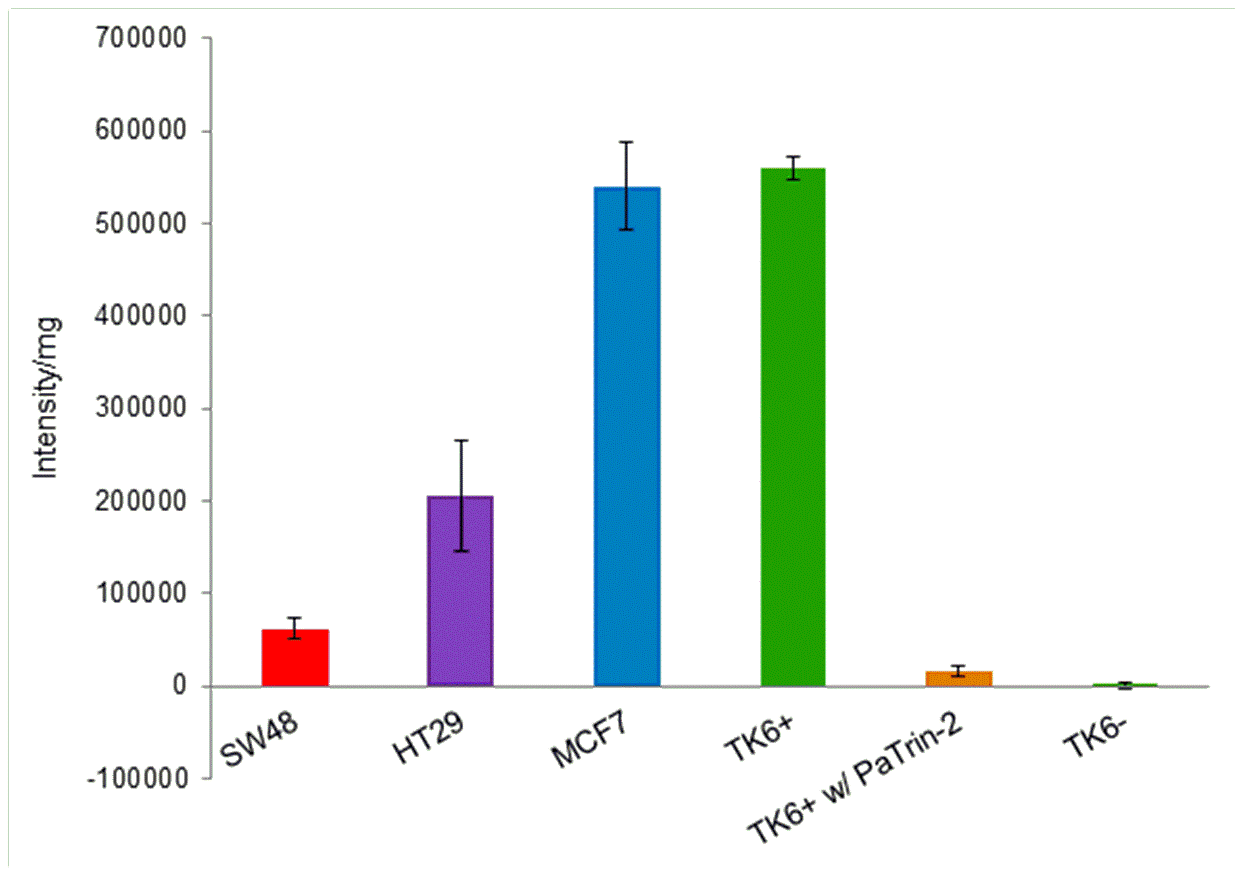


**Figure G. Final fluorescence changes in extracts.** Bar graph representation of final fluorescence changes measured by NR-1 in cell extracts (according to Fig 4 and Fig 5). Single intensity at 520 nm was obtained after a plateau in fluorescence signal change was observed. The intensity was subtracted from background probe fluorescence (probe alone spectrum) and normalized by total mg of protein. Final probe concentration was 50 nM and data was acquired at 37 °C. Each measurement was performed in triplicate. Error bars represent standard deviation.


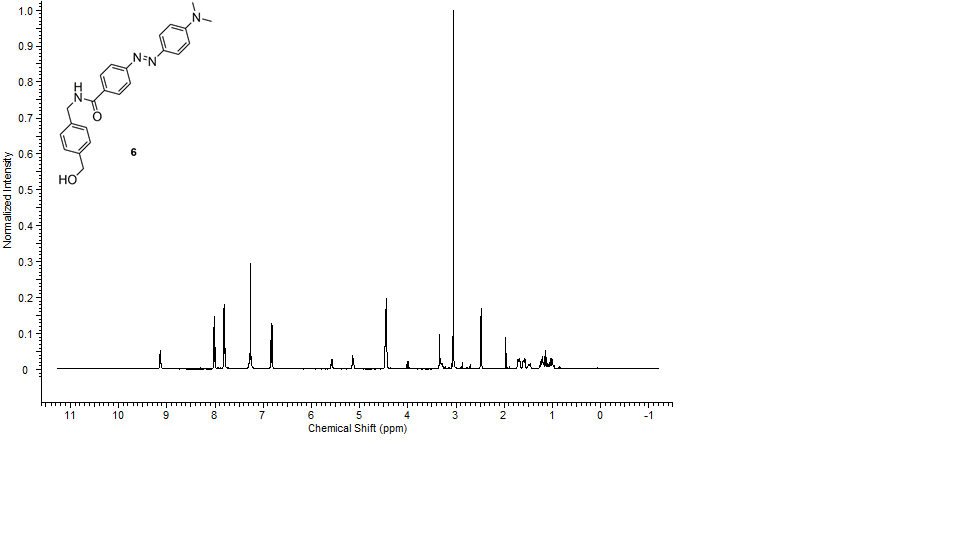

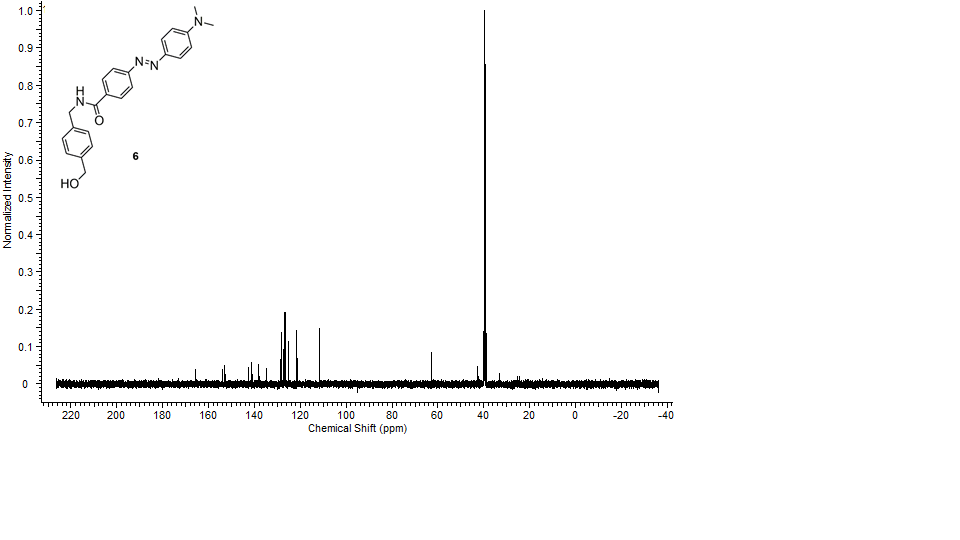

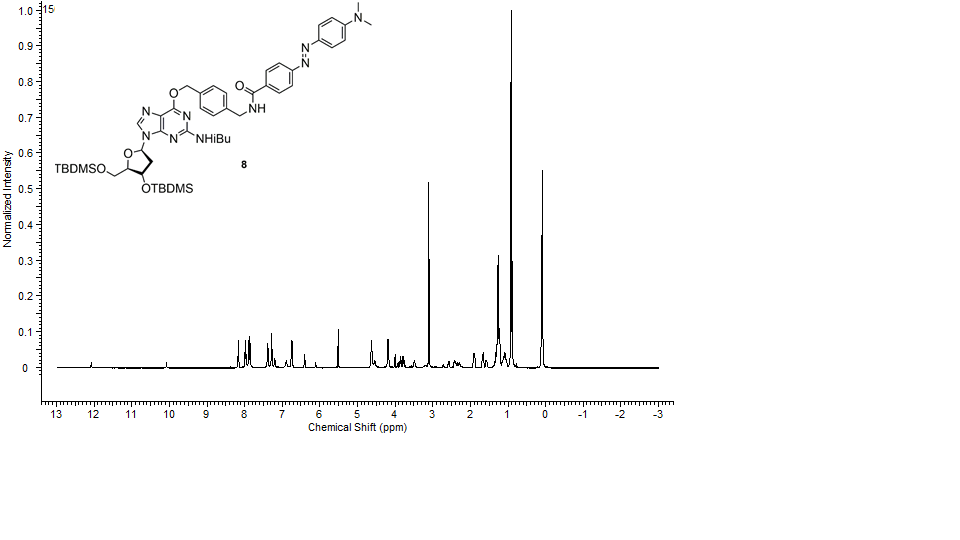

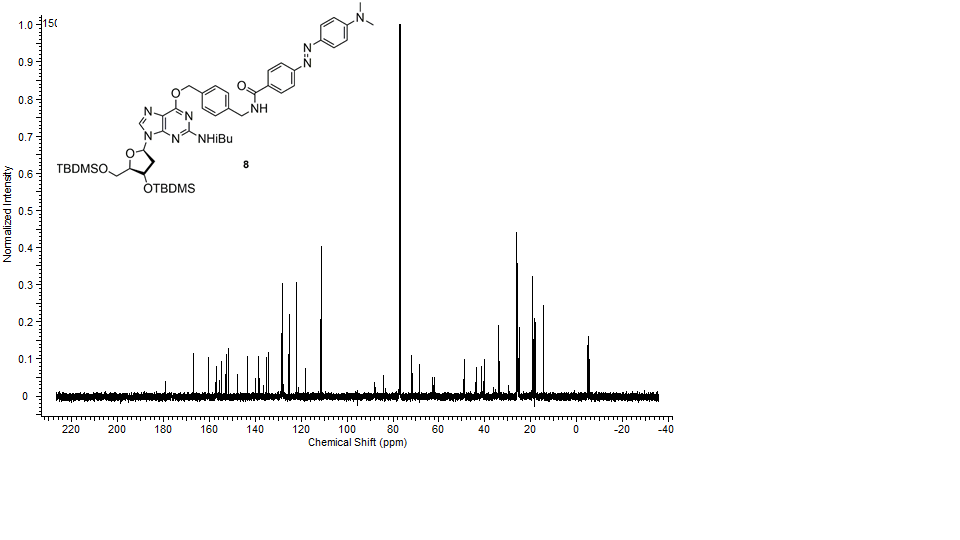

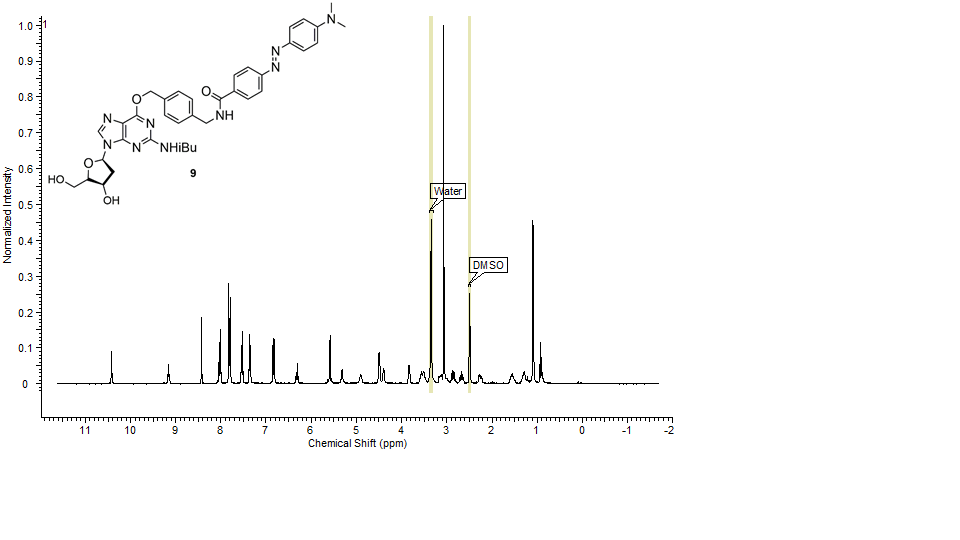

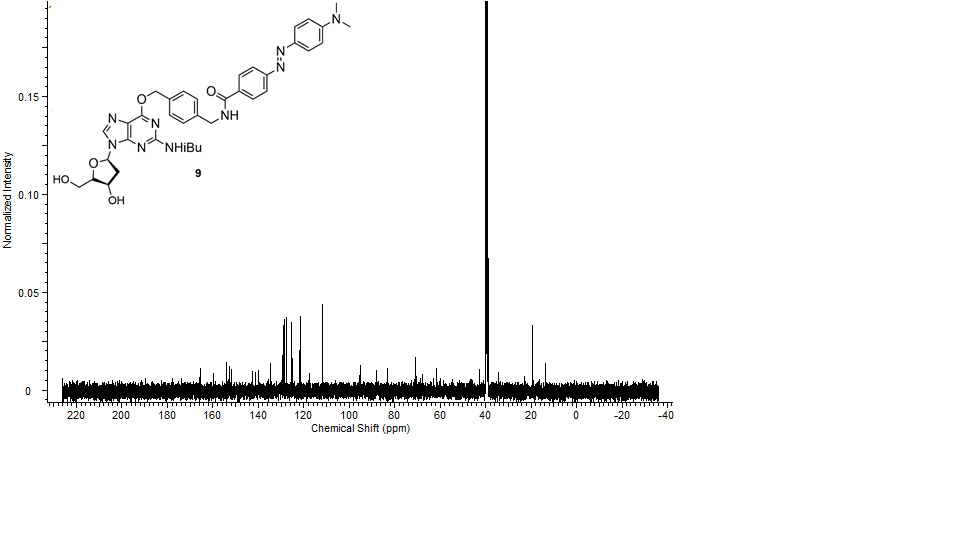


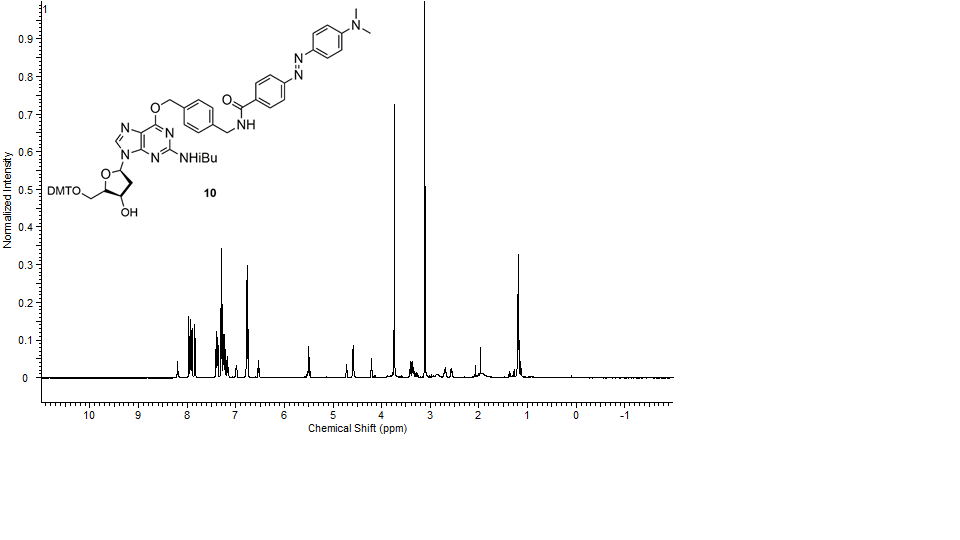

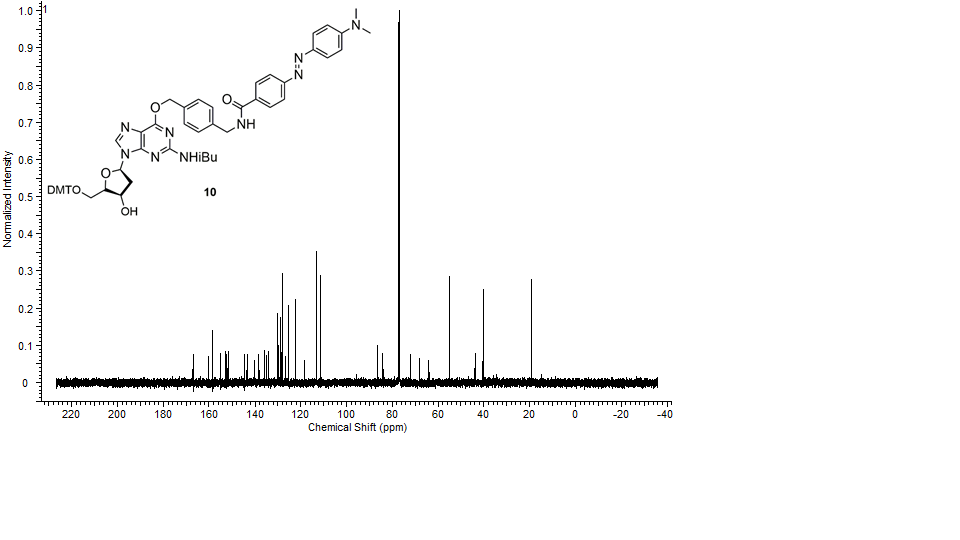


**References**

Gao J, Strässler C, Tahmassebi D, Kool ET. Libraries of composite polyfluors built from fluorescent deoxyribosides. J Am Chem Soc. 2002;124:11590–1.
